# The role of ASF1B in disease pathogenesis and treatment: from histone chaperone to clinical target

**DOI:** 10.1080/15384047.2026.2755094

**Published:** 2026-11-03

**Authors:** Ying Deng, Hao Xie, Yao Chen, Rong Zhang, Qingrong Yue, Yongkang Wu

**Affiliations:** a Department of Urology, West China Hospital Sichuan University Jintang Hospital·Jintang First People's Hospital, Chengdu, China; b Department of Radiology, West China Hospital Sichuan University Jintang Hospital·Jintang First People's Hospital, Chengdu, China; c Department of Emergency, West China Hospital Sichuan University Jintang Hospital·Jintang First People's Hospital, Chengdu, China; d Department of Nursing, West China Hospital Sichuan University Jintang Hospital·Jintang First People's Hospital, Chengdu, China; e Department of Laboratory Medicine, West China Hospital, Sichuan University, Chengdu, China; f West China Hospital Sichuan University Jintang Hospital·Jintang First People's Hospital, Chengdu, China

**Keywords:** ASF1B, histone chaperone, prognostic biomarker, cancer, epigenetics, cell cycle

## Abstract

ASF1B is a critical molecular chaperone for histones H3 and H4 and functions as a core regulator of chromatin assembly, DNA replication, and repair. In recent years, multiple studies have revealed that ASF1B is abnormally overexpressed in various malignant tumors and is correlated with disease progression and poor prognosis, demonstrating its potential as a prognostic biomarker across different cancer types. ASF1B drives tumor cell proliferation, metastasis, treatment resistance, and immune evasion through multiple mechanisms, including direct regulation of the cell cycle, participation in DNA damage repair, and remodeling of the tumor immune microenvironment. Besides, its potential role in non-cancerous diseases has also begun to attract attention. This article systematically reviews the biological characteristics of ASF1B, its expression patterns and functional mechanisms in disease, and explores its translational clinical prospects as a diagnostic marker and therapeutic target, aiming to provide new insights for the precision treatment of related diseases.

## 1. Introduction

In eukaryotic cells, the interpretation, replication, and transmission of genetic information rely on the dynamic assembly and remodeling of chromatin, a process precisely regulated by histone molecular chaperones. The Anti-Silencing Functional Protein 1 (ASF1) family has attracted significant attention as a pivotal hub in histone H3-H4 metabolism.^1^ In vertebrates, ASF1A and ASF1B originated from an ancient gene duplication event, and both have undergone functional specialization during evolution. Structural differences determine their different downstream interaction selectivity, making ASF1B's function closely related to the process of cell proliferation.^2^ At the molecular level, ASF1B is a core executor of chromatin assembly during DNA replication in the S phase. It coordinates histone delivery directly at the replication fork by binding to the replication helicase MCM2-7, ensuring immediate nucleosome assembly of newly synthesized DNA.^3^ This process is regulated by phosphorylation of Tousled-like kinases (TLKs) to enhance their histone transfer efficiency.^1^ ^,^ ^4^ In addition, ASF1B is involved in DNA damage repair. It can be recruited to double-strand breaks by the 53BP1-RIF1 pathway and guides non-homologous end joining repair by promoting local heterochromatinization tendency.^5^ This establishes a connection between genomic stability and therapeutic resistance. Due to ASF1B's central role in maintaining genomic integrity in proliferating cells, its dysfunction is closely linked to the occurrence and progression of cancer. ASF1B is abnormally overexpressed in various malignant tumors and is associated with poor prognosis.^1^ ^,^ ^6^ ^,^ ^7^ Its carcinogenic mechanism is multifaceted: it not only promotes cell proliferation, but also affects genomic stability and therapeutic response, and can reshape the tumor immune microenvironment. Moreover, interfering with ASF1 function can induce alternative lengthening of telomeres, supporting interest in ASF1B as a therapeutic target.^8^

Therefore, ASF1B has become a key molecule that connects basic biology to clinical translation. This article aims to systematically review its biological characteristics and mechanisms of action in disease (especially cancer), and to explore its translational prospects as a diagnostic marker and therapeutic target.

## 2. Biological characteristics of ASF1B

The functional diversity of ASF1B is rooted in its unique molecular structure and achieved through precise regulation of core cellular processes. An in-depth analysis of its various aspects, from structure to function, is the basis for understanding its role under physiological and pathological conditions.

### 2.1. Molecular structure and functional domains

The functional specificity of ASF1B originates from its evolutionary and structural basis. The ASF1B gene, located at 19p13.12, encodes a histone H3–H4 chaperone that supplies histones to nucleosome assembly sites.^9^ Evolutionary analysis indicates that ASF1B and its paralog ASF1A originated from an ancient gene duplication event, followed by the relocation of ASF1A into an intronic region of *MCM9*. The two genes thus evolved under distinct genomic environments—a “genomic context-driven adaptive conflict,” in which different regulatory constraints on the two copies favored their divergence—which ultimately drove their subfunctionalization. In trypanosomes, the two paralogs show differentiated subcellular localization (cytoplasm and nucleus) and regulate different stages of the cell cycle.^2^ ^,^ ^10^ Functionally, ASF1B is a core executor of DNA replication-coupled chromatin assembly. Yeast complementation experiments have confirmed that human ASF1B can effectively rescue the growth defects caused by *Asf1* deletion and specifically respond to replication stress, with stronger conservation than ASF1A.^11^ ^,^ ^12^ Structurally, differences in the N/C-terminal sequences between the two determine their specific affinity for downstream interacting proteins, such as the histone cell cycle regulator (HIRA) complex.^13^ ASF1B exerts its function through a dynamic network: it participates in histone H3-H4 nuclear import,^14^ maintains histone pool stability with nuclear autoantigenic sperm protein (sNASP),^15^ and passes histones to partners such as retinoblastoma‑associated protein 48 (RbAp48) to complete the assembly.^16^ The activity of this network is precisely regulated, such as being phosphorylated by TLK kinase to promote DNA damage repair.^17^ It can also be specifically inhibited by factors such as CDAN1.^18^ This network establishes ASF1B as a central hub connecting chromatin assembly and DNA metabolism.

### 2.2. Role of ASF1B in the cell cycle

ASF1B is a core cell-cycle factor, particularly in the S phase. Its core function lies in coupling DNA replication and chromatin assembly. It directly binds to the DNA replication helicase MCM2-7, anchoring histone supply at the replication fork, thereby ensuring timely encapsulation of newly synthesized DNA and maintaining high-fidelity replication and genomic stability.^3^ In addition to mediating the deposition of new histones, ASF1B also coordinates the recycling and reuse of parental histones under replication stress. ASF1B maintains the continuity of epigenetic information by temporarily storing and rapidly recalling histones with specific pre-deposition modifications.^19^ Current evidence suggests that the deposition of both new and old histones relies on ASF1A/B, which are indispensable for the effective incorporation of histone variants H3.1 and H3.3.^20^ ASF1B also participates in maintaining genomic end stability: ASF1A and ASF1B jointly inhibit the alternative lengthening of telomeres (ALT) pathway; simultaneous loss of both functions can induce ALT characteristics, revealing their synergistic role in telomere stability.^8^ This function is highly conserved evolutionarily. For instance, the homolog kinase TLK1 in trypanosomes coordinates DNA replication and mitosis processes through phosphorylation of ASF1A/B, further confirming the conservation and core status of this pathway.^21^

### 2.3. Transcriptional regulation and post-translational modification

The expression and activity of ASF1B are regulated by both transcriptional and post-translational modifications (Figure 1). At the transcriptional level, ASF1B is directly activated by the cell cycle core transcription factor E2F1, thereby integrating its expression into the cell proliferation signaling network.^22^ At the post-translational level, Tousled-like kinase-mediated phosphorylation is crucial for regulating its activity. The highly active TLK in the S phase significantly enhances its binding affinity with histone H3-H4 dimers by specifically phosphorylating the C-terminus of ASF1B and promotes its interaction with the downstream assembly factor chromatin assembly factor 1 (CAF‑1), thereby “activating” its histone transfer function.^4^ ^,^ ^23^ This process is subject to feedback regulation by DNA replication checkpoints: when replication is blocked, TLK activity is rapidly inhibited, leading to dephosphorylation of ASF1B and synchronizing the rate of chromatin assembly with the replication process.^24^ Beyond modulating activity, TLK-mediated phosphorylation stabilizes ASF1 proteins against proteasomal degradation, adding a second layer of post-translational control.^25^ The absence of TLK function directly impairs histone H3.1 and H3.3 deposition, thereby providing reciprocal evidence for the centrality of this regulatory axis.^26^

**Figure 1.**
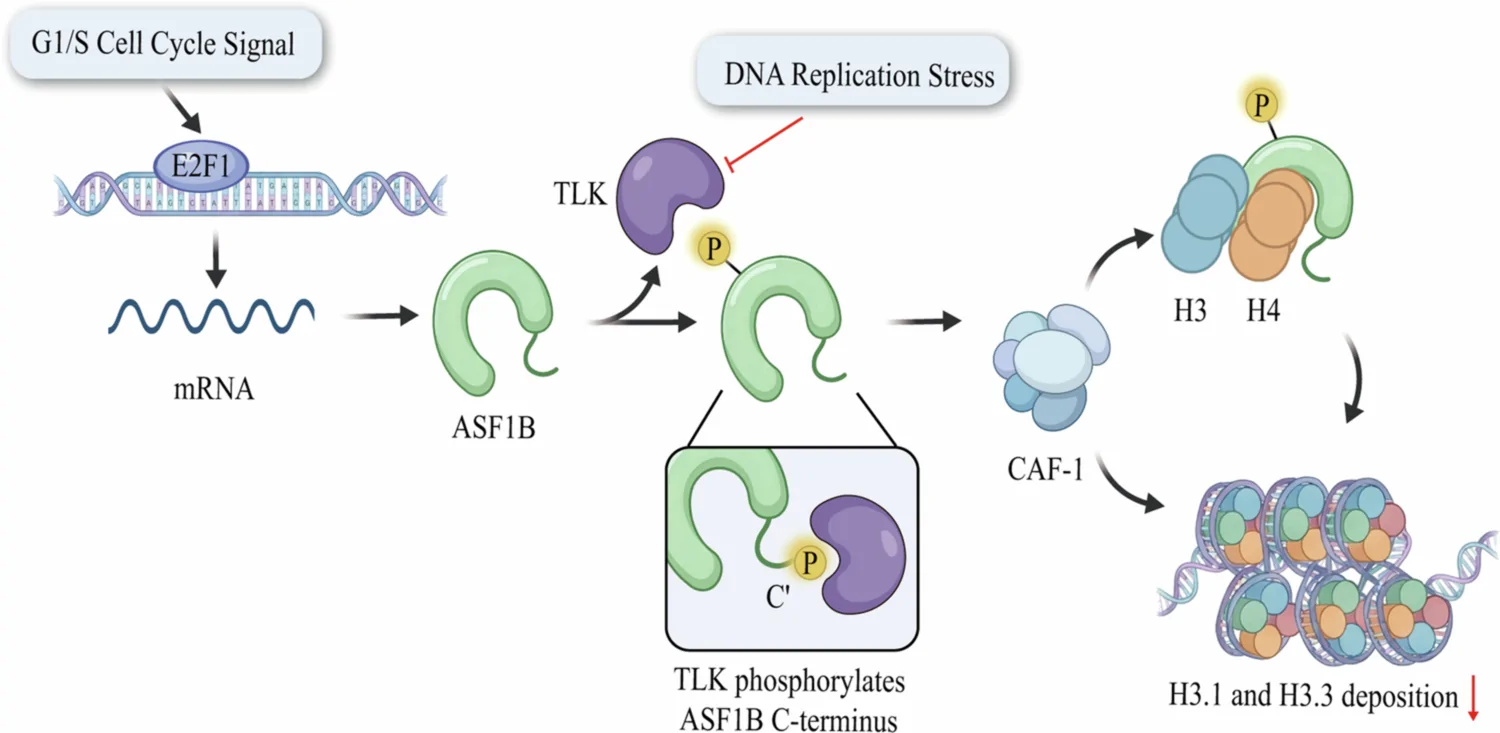
Transcriptional and post-translational regulation of ASF1B. G1/S cell cycle signals activate the transcription factor E2F1, which binds to the *ASF1B* promoter to drive its transcription and subsequent protein synthesis. The resulting ASF1B protein is phosphorylated at its C-terminus by Tousled-like kinases (TLK), which promotes its binding to histone H3-H4 dimers and facilitates the deposition of the histone variants H3.1 and H3.3 via the downstream chaperone CAF-1. DNA replication stress feeds back to inhibit TLK activity, providing a regulatory checkpoint that couples chromatin assembly with replication fork progression.

### 2.4. The mechanism of action of ASF1B in cellular signaling pathways

ASF1B has a wide range of functions involving multiple cellular signaling pathways. In DNA damage repair, ASF1B can be recruited to double-strand breaks by the 53BP1-RIF1 pathway, guiding repair towards non-homologous end joining by promoting heterochromatinization of distal chromatin.^5^ In cell differentiation, it assists in activating myogenic genes as part of the HIRA complex.^27^ In terms of metabolic regulation, ASF1B can specifically promote the proliferation of human pancreatic *β* cells, providing a new strategy for diabetes cell therapy.^28^ ^,^ ^29^ Under conditions of cellular stress, ASF1B works synergistically with HIRA to drive global transcriptional reprogramming after UV damage.^30^ In addition, it also sensitizes cells to proteasome-inhibitor-induced death, as identified in a genome-wide siRNA screen,^31^ though the mechanistic basis is unclear.

Interestingly, various viruses can hijack ASF1B to facilitate their own lifecycles; for instance, herpesviruses recruit ASF1B to viral replication compartments via the HCF-1 protein.^32^ Similarly, the EB virus protein BKRF4 can form a complex with ASF1B to jointly bind to histone proteins.^33^ These functions make ASF1B an important therapeutic target. Previous studies have identified small-molecule compounds that can specifically inhibit ASF1 and have verified their antiviral effects.^34^

### 2.5. Diversity of ASF1B function and evolutionary perspective

The functional diversity of ASF1B has long been evident in evolution. Even in plants such as *Arabidopsis thaliana*, ASF1B exhibits a complex functional profile: it is regulated by the cell cycle transcription factor E2F and involved in UV-induced DNA damage repair.^35^ During the heat stress response, it activates specific gene transcription by promoting nucleosome removal and histone H3K56 acetylation.^36^ In addition, ASF1B shares functional redundancy with ASF1A, where their combined activity is indispensable for key reproductive processes such as gametophyte development and fertilization.^37^ These studies not only reveal the central role of ASF1B in regulating chromatin dynamics during basic life processes but also suggest that genetic intervention (such as gene insertion) on its function may induce complex phenotypes that are not caused by single-gene loss.^38^

In the reproductive system of higher mammals, ASF1B is a core factor regulating cell reprogramming. It is highly expressed in human, rhesus monkey, and mouse oocytes, driving epigenetic remodeling by relaxing chromatin structure, thus laying the foundation for zygotic genome activation.^39^ Its expression is precisely regulated by temporal and spatial cues. For instance, during the critical transition period of early embryonic development in pigs, the length of its mRNA's 3' untranslated region undergoes specific changes.^40^ The dysregulation of this regulatory mechanism has been associated with reproductive aging and exposure to environmental toxins. In this respect, ASF1B is abnormally upregulated in aging oocytes, which is related to epigenetic disorders and abnormalities in the cell cycle.^41^ The environmental toxin monobutyl phthalate can induce abnormal expression, thereby impairing epigenetic homeostasis and embryonic developmental potential.^42^ This evidence establishes the crucial role of ASF1B in preserving gamete quality and early embryo programming, consistent with a broad role in chromatin regulation during cell-fate transitions. Beyond reproduction, ASF1B occupies the majority of H3.3 nucleosomes in erythroid cells, determining H3.3 enrichment at erythroid gene promoters and enhancers^43^; accordingly, its loss perturbs globin gene expression balance, highlighting a therapeutic angle in *β*-hemoglobinopathies.^43^ ^,^ ^44^

In summary, the same ASF1B activity that supports normal cell proliferation can, when dysregulated, promote disease. Under physiological conditions, E2F1-driven transcription and TLK-mediated phosphorylation couple histone supply to DNA replication and repair. Under pathological conditions—overexpression, or hijacking by viruses—this coupling fails, producing the genomic instability that underlies tumorigenesis. Whether ASF1B inhibition can exploit this failure with an acceptable therapeutic window is a question for the preclinical studies discussed in Section 4.

## 3. The role of ASF1B in the pathogenesis and progression of diseases

The core function of ASF1B is to coordinate chromatin dynamics and cell fate transitions, and its functional deficiency directly leads to developmental failure.^45–47^ Overall, ASF1B promotes disease (notably cancer) by subverting its normal regulatory functions, thereby disrupting essential chromatin dynamics, including DNA replication, repair, cell cycle control, and epigenetic stability. In cancer, the disorder of these processes is “hijacked” and amplified, driving malignant phenotypes such as uncontrolled proliferation, immune microenvironment remodeling, and metastasis.

### 3.1. The role of ASF1B in malignant tumors

The central role of ASF1B in cancer stems from its function as a central regulator of chromatin dynamics. Its pro-cancer mechanisms are not singular but rather form a multifaceted carcinogenic network. These mechanisms operate across malignancies with high global burden, including lung, pancreatic, and liver cancers.^48‐50^ First, this molecule promotes cancer through four core mechanisms within tumor cells: (1) driving cell cycle progression and proliferation; (2) activating key oncogenic signaling pathways; (3) mediating genomic instability and treatment resistance; and (4) reshaping the tumor immune microenvironment. Second, these functions are precisely regulated by an upstream regulatory network. Finally, several additional cancers do not yet fit this framework. Their evidence is either too preliminary to assign to a mechanism, or their clinical context (hormone signaling, partner histone variants, copy-number amplification) lies outside this framework. These are gathered separately in Section 3.1.6. In vivo evidence from across this section is consolidated in Table 1, and Figure 2 integrates the four core mechanisms, the upstream regulatory network, and the cancer-type-specific evidence into a single framework.

**Figure 2.**
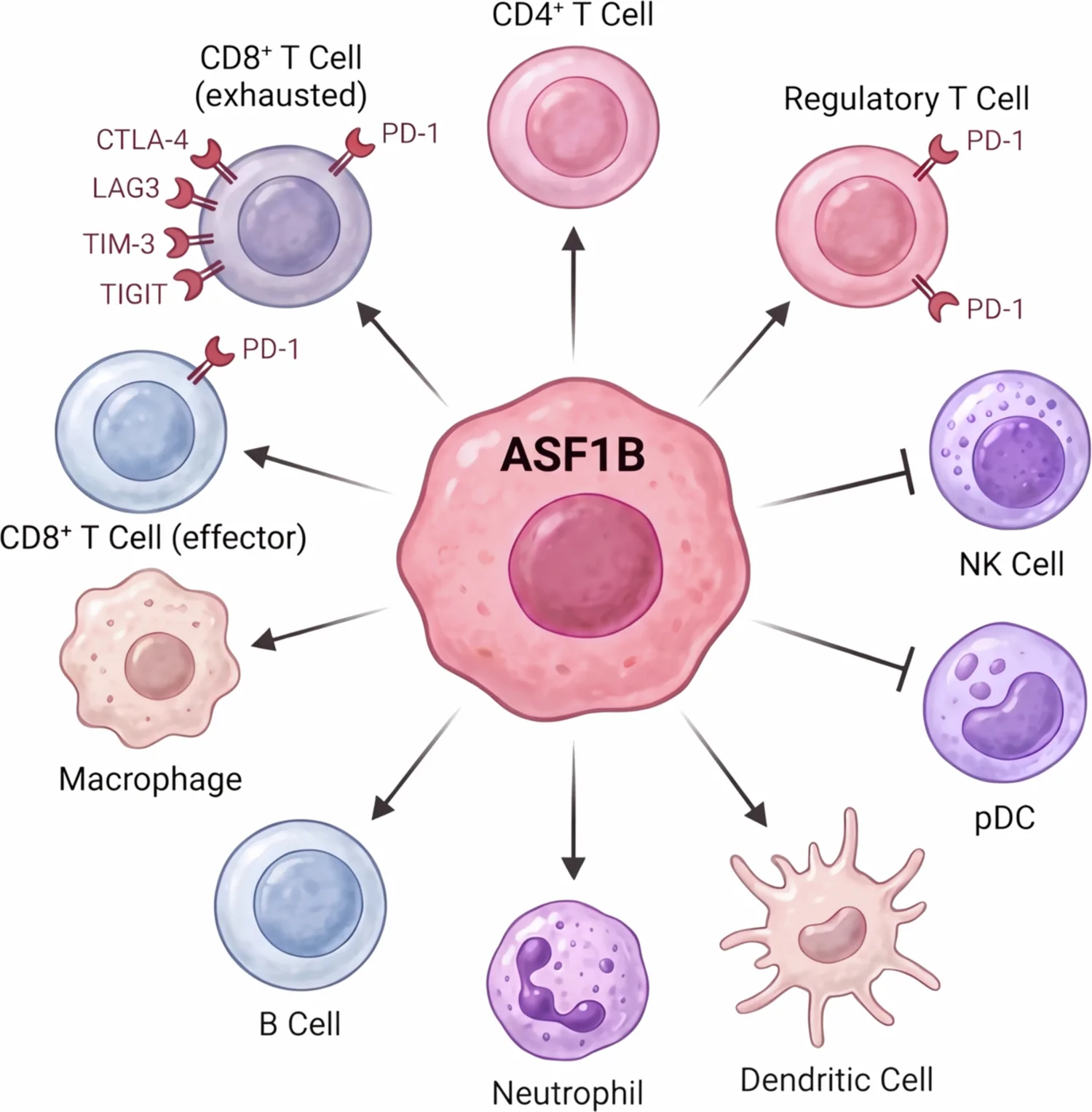
Multidimensional regulatory network of ASF1B in cancer. The diagram depicts ASF1B as a core molecular switch integrating upstream regulatory inputs and driving multiple downstream malignant phenotypes. On the left, At the top, the upstream regulatory network comprises non-coding RNAs (circRNA, miRNA) and transcription factors that collectively control ASF1B expression. In the center, ASF1B functions as a hub to transduce these signals. On the right, Around the central hub, ASF1B orchestrates a broad spectrum of cancer-relevant processes, including cell cycle progression, oncogenic signaling (e.g., PI3K/AKT, Myc), metastasis, survival/anti-apoptosis, genomic instability, drug resistance, and immune evasion. These interconnected pathways collectively promote tumor growth and remodel the tumor immune microenvironment.

#### 3.1.1. ASF1B as a driver of cell cycle progression and proliferation

ASF1B's basic oncogenic role is to sustain cell cycle progression and proliferation, an extension of its physiological function of supplying histones H3–H4 for replication-coupled chromatin assembly. Cancer cells appear to hijack this process to support their unlimited proliferation. The pro-proliferative function is conserved across multiple solid tumors and hematological malignancies, although the strength of evidence varies considerably.

Three downstream routes appear to mediate ASF1B-driven proliferation. The best-characterized is the CDK9/cyclin B1 route. In cervical cancer, ASF1B forms a stable complex with CDK9 and promotes its stabilization, thereby facilitating S-phase entry.^51^ This study also provides one of the few complete evidence chains in the field, coupling the molecular mechanism with xenograft validation in which ASF1B knockdown suppressed tumor growth and induced apoptosis. In HCC, ASF1B positively regulates PCNA and cyclin B1, and its proliferative effect correlates with high expression of AURKB and CCNB1; high ASF1B expression is an independent marker of poor prognosis.^52‐54^ Whether HCC operates through the same CDK9-dependent mechanism as cervical cancer, however, remains untested.

A second, kinase-coupled route involves the TLK–ASF1B axis. In glioma, ASF1B directly interacts with TLK1 to form a functional axis that jointly drives tumor proliferation and migration.^55^ In parallel, CIA-II (the ASF1B paralog) has been identified as an independent prognostic factor in lower-grade glioma, with in vitro studies confirming its proliferation-promoting role.^56^ The relevance of this axis extends beyond solid tumors: in AML, ASF1B overexpression accelerates leukemia progression in mouse models, whereas *Asf1b* or *Tlk2* deficiency delays disease progression.^57^ These findings position TLK kinases not merely as post-translational activators of ASF1B but as druggable upstream nodes of its oncogenic activity.

A third route, distinct again, operates in LUAD through the p53/EMT axis: ASF1B upregulation decreases P53 and P21 while increasing Snail and Slug. Pharmacological rescue experiments support causality: activating P53 with Nutlin3 attenuates the EMT-promoting effect of ASF1B, whereas inhibiting P53 with pifithrin-*α* promotes EMT and counteracts the effect of ASF1B knockdown.^58^ The same study provides in vivo validation: ASF1B knockdown inhibited xenograft tumor growth and reduced Ki-67 expression.^58^ Clinically, high ASF1B expression is associated with poor overall and progression-free survival in LUAD patients.^59^ In breast cancer, the picture is thinner: ASF1B is highly expressed in proliferative subtypes (luminal B and HER2-positive) and is associated with poor prognosis, but the evidence remains primarily computational and tissue-based.^60^ ^,^ ^61^ Similar caveats apply to bladder cancer, where associations with core proliferation processes have been established, but mechanistic and in vivo evidence is lacking.^62^ Gastric cancer falls between these tiers: loss of ASF1B suppresses subcutaneous xenograft growth in mice, but the downstream mechanism has not been defined.^63^

Whether these routes operate in parallel or are selected by tumor type could be tested directly: knockdown of ASF1B across cell lines with intact or disrupted CDK9, TLK1, or p53 function (via genetic depletion or pharmacological inhibition) from different cancer types would reveal which downstream dependency dominates in each context.

#### 3.1.2. ASF1B-mediated activation of key oncogenic signaling pathways

Beyond directly driving the cell cycle, ASF1B amplifies its oncogenic effects by engaging several classical signaling pathways.

The most frequently implicated route is the PI3K/AKT/mTOR axis. In gastric cancer, ASF1B activates PI3K/AKT/mTOR signaling to drive tumor progression, and its overexpression is an independent poor prognostic factor.^63^ In colorectal cancer, the anti-tumor effect of ASF1B silencing is partially reversed by PI3K/AKT pathway activators.^64^ This study coupled clinical tissue validation (qRT-PCR and IHC) with in vitro assays and xenograft mouse models, confirming that ASF1B silencing suppresses tumorigenesis and that PI3K activation partially restores malignant phenotypes.^64^ In ccRCC, ASF1B promotes proliferation and migration through AKT/P70 S6K1 signaling, and AKT inhibitor blocked this effect in vitro.^65^ In the same cancer, ASF1B has additionally been nominated as a candidate for drug repositioning on the basis of its prognostic association,^66^ although this translational potential awaits experimental confirmation. Collectively, these studies establish PI3K/AKT/mTOR as a recurrent downstream effector of ASF1B; however, whether ASF1B engages this pathway directly or secondarily through chromatin-mediated transcriptional programs has not been resolved in most contexts.

A second set of mechanisms operates through transcriptional and epigenetic control of oncogenic programs. In pancreatic cancer, ASF1B increases H3K56 acetylation in a CBP-dependent manner, promoting H3K56ac at the c-Myc promoter and thereby enhancing c-Myc expression; CBP and c-Myc inhibitors reverse the pro-tumor effects of ASF1B.^67^ In gastric cancer, currently the hub of evidence for this axis, the transcription factor FOXM1 directly binds the ASF1B promoter and drives its expression, while ASF1B in turn regulates the mitochondrial protein PRDX3 to promote proliferation and redox balance.^68^ More recently, ASF1B was shown to downregulate the histone variant H2AC20, thereby activating PI3K/AKT and ERK1/2 signaling in gastric cancer; ASF1B knockdown increased CD8^+^ T-cell infiltration in mouse models and organoids.^69^ Additionally, ASF1B activates Myc-targets-v1 and Myc-targets-v2 pathways in gastric cancer, and silencing ASF1B reduces Myc, MCM4, and MCM5 expression, thereby inhibiting proliferation, invasion, and cisplatin resistance.^70^ This Myc-axis finding complements the CBP/H3K56ac/c-Myc mechanism described in pancreatic cancer, suggesting that Myc activation is a recurring downstream event of ASF1B. The H2AC20 mechanism converges on the same PI3K/AKT axis described above, suggesting that the “signaling” and “epigenetic” routes are interconnected rather than parallel: ASF1B appears to feed pathway activation at multiple levels, from histone supply to histone modification to pathway crosstalk. In triple-negative breast cancer, ASF1B also confers cisplatin resistance through this pathway,^71^ and its therapeutic implications are discussed in Section 4.2.

The strength of evidence again varies by cancer type. Gastric cancer possesses the most complete evidence chain in this section—clinical association, defined molecular mechanisms, and in vivo validation including immune readouts. Colorectal cancer now also has clinical tissue, in vitro, and in vivo validation.^64^ Pancreatic cancer provides mechanistic depth for the CBP/H3K56ac/c-Myc axis with pharmacological rescue, though whether this mechanism extends to other RAS-driven tumors is unknown. For ccRCC, the evidence remains largely in vitro and computational, and the upstream events linking ASF1B to PI3K/AKT activation have not been defined.

A direct test would be to check whether PI3K/AKT phosphorylation changes within minutes to hours of acute ASF1B depletion (direct consequence) or only after prolonged knockdown (secondary adaptation). The same uncertainty applies to the epigenetic mechanisms documented in pancreatic and gastric cancers: they may be specific to those settings, or simply underexplored elsewhere. The distinction matters in practice. If specific, ASF1B inhibition would need to be paired with pathway-specific inhibitors by cancer type; if general, the epigenetic layer could become a shared target across tumors.

#### 3.1.3. ASF1B in genomic instability and treatment resistance

Because ASF1B maintains genomes through histone metabolism and DNA replication, its dysregulation in cancer plausibly feeds into DNA damage response (DDR) and treatment resistance. ASF1B's physiological role predicts a link to replication stress and chromosomal instability, but direct evidence in these areas is lacking; the available literature concentrates on DDR and chemoresistance.

The most direct mechanistic links run through DDR and chromatin maintenance. ASF1B is recruited to DNA double-strand breaks (DSBs) by the 53BP1–RIF1 pathway, where it promotes local heterochromatinization and steers repair toward non-homologous end joining (NHEJ).^5^ This function connects ASF1B to therapeutic resistance conceptually, as NHEJ is a major mechanism of resistance to DNA-damaging agents, though this link has not yet been tested by combining ASF1B inhibition with DNA-damaging therapy in the same study. A related but distinct mechanism operates in ccRCC, where SETD2 loss creates a permissive epigenetic landscape that promotes metastasis; targeting ASF1A/ASF1B complexes reduces distant metastasis in xenograft models.^72^ Importantly, this study nominates the ASF1 family—rather than ASF1B alone—as a druggable vulnerability in an epigenetically defined tumor context, and its relevance to genome stability is indirect. A second, mechanistically defined link runs through ferroptosis: in gastric cancer, ASF1B recruits HOXB3 to drive ZDHHC9 transcription; ZDHHC9 palmitoylates PCBP1, stabilizing SLC7A11 and suppressing ferroptosis, thereby promoting liver metastasis in a spleen-injection model (in vivo).^73^ This axis also correlated with PD-L1 expression, connecting metastatic regulation to immune evasion. A third mechanism involves checkpoint control. In pancreatic cancer, ASF1B silencing activates the checkpoint kinases Chk1 and Chk2, leading to S-phase arrest, increased DNA damage, and enhanced cisplatin sensitivity.^74^ This positions ASF1B as a candidate biomarker for platinum-based therapy. However, the evidence remains in vitro, and no animal study has yet confirmed that ASF1B inhibition sensitizes pancreatic tumors to cisplatin in vivo.

Functional evidence for a role in chemoresistance is strongest in bladder cancer, where ASF1B suppression reduces cisplatin resistance and inhibits tumor growth in mouse models^62^—one of the few settings in which this role has been validated in vivo. The evidence in locally advanced rectal cancer is weaker still: high ASF1B expression has been associated with resistance to neoadjuvant chemoradiation, but this evidence is entirely computational and awaits prospective validation.^75^ The cisplatin-resistance phenotypes in gastric cancer (via the Myc pathway)^70^ and triple-negative breast cancer (where the circUBAP2/miR-300 axis upregulates ASF1B to activate PI3K/AKT/mTOR signaling)^71^ provide additional mechanistic routes; as discussed in Section 3.1.2, these converge with ASF1B's signaling functions rather than representing a separate resistance program. Which of these mechanisms dominates ASF1B-dependent resistance in any given tumor remains unknown; beyond the bladder cancer proof of concept, combining ASF1B inhibition with DNA-damaging agents has yet to be tested in vivo in other tumor types.

#### 3.1.4. ASF1B-mediated remodeling of the tumor immune microenvironment

Among ASF1B's oncogenic functions, its role in the tumor immune microenvironment (TIME) is the least mechanistically defined: the evidence remains overwhelmingly computational, and “immune infiltration” should not be treated as a monolithic pro-tumor process, since different immune populations can exert opposing functions.

At the level of immune cell infiltration, a recurring pattern emerges: ASF1B expression is positively correlated with the infiltration of multiple immune cell types, yet this apparent “immune-rich” state is paradoxically associated with poor prognosis. In HCC, computational deconvolution of TCGA data suggests that ASF1B is positively correlated with CD4^+^ and CD8^+^ T cells, B cells, macrophages, neutrophils, and dendritic cells, as well as with regulatory T cell (Treg) infiltration.^76^ A separate HCC study independently confirmed these infiltration correlations and further linked ASF1B to immune infiltration networks through co-expression analysis.^77^ The co-occurrence of effector T cells (CD8^+^) and suppressive populations (Tregs) suggests that ASF1B may not simply suppress immunity, but rather reshape the balance between immune activation and exhaustion. At single-cell resolution, ASF1B was identified among nine genes defining an immunosuppressive CD4^+^ T-cell subtype in HCC, linking its expression to immune evasion and patient prognosis.^78^

This pattern extends to other cancer types, with cell-type-specific differences. In gastric cancer, computational analysis indicates that high ASF1B expression is associated with increased infiltration of activated dendritic cells, neutrophils, Th2 cells, Th17 cells, and Tregs, alongside decreased B cells, mast cells, and naïve/memory T cell subsets.^79^ In glioma, ASF1B correlates positively with Th2 cells, macrophages, and activated dendritic cells, but negatively with plasmacytoid dendritic cells and NK CD56^bright cells.^80^ In breast cancer, ASF1B is positively correlated with B cells, neutrophils, and dendritic cells, and negatively correlated with macrophage infiltration.^60^ These divergent patterns suggest that ASF1B's impact on immune infiltration is context-dependent rather than uniform.

Mechanistically, the most direct evidence linking ASF1B to immune evasion involves immune checkpoint regulation. In gastric cancer, ASF1B expression is significantly correlated with tumor mutational burden, microsatellite instability, PD-1 immunophenoscore, and the expression of PD-1, PD-L1, CTLA-4, LAG3, and HAVCR2.^79^ At the functional level, in thyroid cancer, ASF1B knockdown reduces FOXP3 expression in vitro, and FOXP3 overexpression restores the migration and invasion abilities suppressed by ASF1B knockdown, suggesting that ASF1B may promote Treg recruitment through FOXP3.^81^ In HCC, experimental evidence further shows that ASF1B knockdown reduces the expression of CD86, CD8, STAT1, STAT4, CD68, and PD-1, and flow cytometry confirmed that SNHG3 promotes PD-1 expression through ASF1B.^53^ In NSCLC, ASF1B ablation induced p53-related ferroptosis, reduced CD8^+^ T-cell apoptosis, increased IL-2 and IFN-*γ* release, and lowered PD-L1/PD-1 expression in tumor-PBMC co-cultures (in vitro), and enhanced the efficacy of anti-PD-L1 therapy in a syngeneic mouse model (in vivo).^82^ In vivo, ASF1B knockdown increased CD8^+^ T-cell infiltration in gastric cancer xenografts and organoids (Section 3.1.2)^69^; together with the NSCLC study above, these findings are the experimental anchors for the hypotheses below and are summarized in Figure 3.

**Figure 3.**
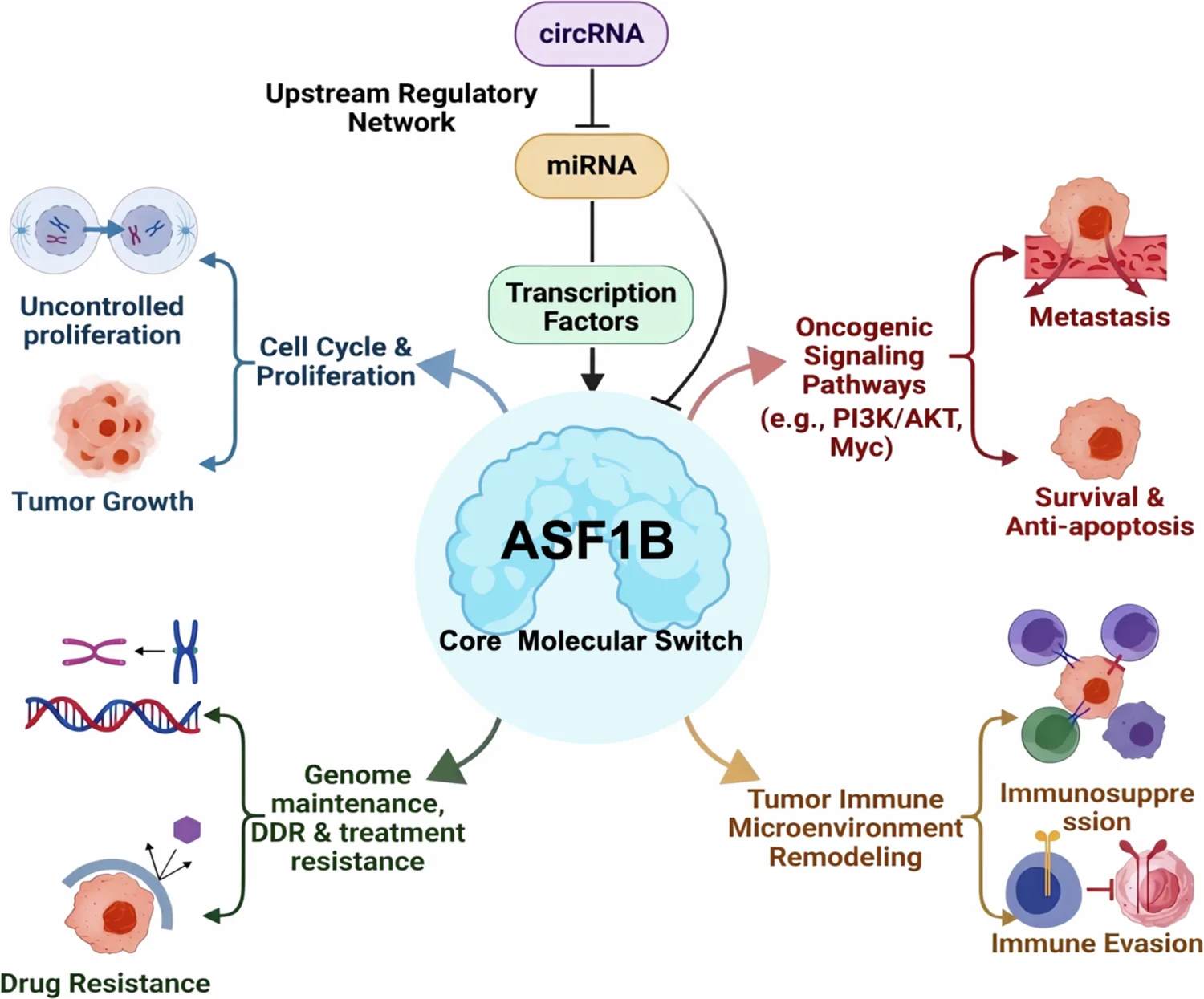
ASF1B in the tumor immune microenvironment. Arrows from the ASF1B-expressing tumor cell denote positive correlations with immune cell infiltration (predominantly computational); blunt-ended lines denote negative correlations (NK cells and plasmacytoid dendritic cells [pDC] in glioma). The two CD8^+^ T cells illustrate the co-existence of effector (single PD-1 tag) and exhausted (multiple checkpoint tags) cytotoxic T cells in high-ASF1B tumors. The experimental evidence (reduced FOXP3 upon ASF1B knockdown in thyroid cancer cells, and the in vivo gastric and NSCLC findings described below) upon ASF1B knockdown in thyroid cancer cells (in vitro), increased CD8^+^ T-cell infiltration upon ASF1B knockdown in gastric cancer xenografts and organoids (in vivo), and enhanced anti-PD-L1 efficacy upon ASF1B ablation in an immunocompetent NSCLC model—together with the clinical associations in treated cohorts (Imvigor210, a urothelial carcinoma cohort serving as cross-cancer reference; combined anti-CTLA-4/PD-1 in gastric cancer; negative association in male thyroid cancer), are summarized here.

The immunotherapy-response data are apparently contradictory but may be partially reconcilable. In HCC, ASF1B expression is strongly correlated with PD-1, CTLA-4, LAG3, TIM-3, and TIGIT in exhausted T cells.^76^ The same study also evaluated the IMvigor210 cohort, a phase II trial of anti-PD-L1 therapy in urothelial cancer rather than HCC, and found higher objective response rates in patients with high ASF1B expression. Similarly, in gastric cancer, the high-ASF1B group benefited significantly from combined anti-CTLA-4/PD-1 treatment, and high ASF1B was associated with better overall survival following immunotherapy.^79^ These findings are consistent with the “immune activation with exhaustion” model: high-ASF1B tumors are inflamed, checkpoint-high, and thus responsive to checkpoint blockade, even though their baseline prognosis is poor. By contrast, in thyroid cancer, ASF1B expression was negatively correlated with anti-PD-L1 response in male patients,^81^ a sex-specific association whose basis is currently unexplained. In rectal cancer, ASF1B-associated resistance to neoadjuvant chemoradiation showed no correlation with immune cell infiltration,^75^ arguing against a universal immune-mediated mechanism and suggesting that cell-intrinsic programs dominate in at least some contexts.

In summary, ASF1B is most consistently associated with increased infiltration of immunosuppressive populations (Tregs and, in most contexts, macrophages), whereas its relationship with effector T cells is context-dependent: positively correlated with CD8^+^ T cells in HCC, but negatively associated with CD8^+^ T cell infiltration in gastric cancer based on in vivo knockdown experiments.

Several limitations of the current evidence should be noted. Nearly all immune infiltration data summarized here derive from computational deconvolution of bulk tumor transcriptomes (TIMER, CIBERSORT, xCell, ssGSEA), which estimate relative cell abundances but cannot establish causality or capture spatial interactions. Only three studies provide functional validation: the FOXP3–Treg axis in thyroid cancer cells (in vitro), CD8^+^ T-cell infiltration in gastric cancer models (in vivo), and ferroptosis-linked immune modulation with sensitization to anti-PD-L1 in NSCLC (in vitro and in vivo). Whether ASF1B actively shapes the immune microenvironment or merely tracks the proliferative state of the tumor, and whether ASF1B inhibition can turn the “activation-with-exhaustion” state into productive anti-tumor immunity, are both still open questions; the CD8^+^ T-cell increase seen upon knockdown in vivo is a foothold, but it needs validation across cancer types. The divergent immunotherapy-response associations, in turn, require prospective cohorts with standardized immune monitoring and sex-stratified analysis. Until then, the direction and magnitude of ASF1B's effects on immune composition remain the least established aspect of its oncogenic functions.

#### 3.1.5. Upstream regulatory network of ASF1B expression: ceRNA and transcription factors

Several layers of regulation drive the abnormal overexpression of ASF1B in cancer; the best studied are ceRNA (competing endogenous RNA) interactions and transcription factor activation. Mapping this network helps explain context-specific ASF1B upregulation and points to indirect therapeutic strategies that bypass the difficulty of targeting ASF1B directly.

The best-characterized layer is the ceRNA network, in which circular RNAs (circRNAs) and long non-coding RNAs (lncRNAs) act as microRNA sponges to relieve miRNA-mediated repression of ASF1B. In cervical cancer, circCDK17 sponges miR-122-5p to upregulate ASF1B and promote proliferation, migration, and invasion; validated in xenografts, it is one of the few ceRNA mechanisms with in vivo support.^83^ In HCC, an H3K18-lactylation-driven LINC02732/miR-1291 axis upregulates ASF1B, which in turn recruits the lactyltransferase p300, forming a positive feedback loop that drives CD44 expression and tumor growth in vivo.^84^ This axis extends the upstream network from RNA regulation to histone modification crosstalk. In ccRCC, hsa_circ_0002024 sponges miR-129-5p to upregulate ASF1B.^85^ In triple-negative breast cancer, circUBAP2 sponges miR-300 to upregulate ASF1B and confer cisplatin resistance (Sections 3.1.2 and 3.1.3).^71^ A parallel lncRNA-based axis is SNHG3/miR-214-3p in HCC, whose immune phenotypes are discussed in Section 3.1.4. ^53^ Notably, miR-214 also appears as a direct regulator of ASF1B in multiple myeloma (MM), where it represses ASF1B (together with PSMD10) to inhibit DNA replication and promote apoptosis; ASF1B expression is significantly downregulated in MM patients.^86^ ^,^ ^87^ This contrasts with its consistent upregulation in solid tumors and suggests that the regulatory network, not the expression level itself, is what varies between contexts. Beyond sponges and direct repression, miRNA regulation has also been documented in pancreatic cancer (miR-24-3p, with clinical tissue validation but no animal data) and melanoma (miR-767-3p, with in vitro and in vivo evidence).^88^ ^,^ ^89^

A second layer involves direct transcriptional activation. E2F1 binds the ASF1B promoter and activates its transcription (specifically for ASF1B, as ASF1A is not similarly induced), integrating ASF1B into the G1/S program and explaining its proliferation-associated expression pattern.^22^ FOXM1, another proliferation-associated factor, similarly drives ASF1B transcription in gastric cancer (Section 3.1.2).^68^ No study has yet tested whether E2F1 and FOXM1 cooperate or act redundantly across cancer types.

The circCDK17 axis has in vivo validation; most other ceRNA axes rest on dual-luciferase reporter and in vitro assays. The E2F1–ASF1B interaction is supported by chromatin immunoprecipitation and promoter analysis without in vivo testing; the melanoma miR-767-3p axis is the only miRNA mechanism with in vivo support. The MM findings, though mechanistically suggestive, derive largely from clinical correlation and await functional confirmation.

#### 3.1.6. Cancers beyond the core mechanisms: emerging evidence

Not every malignancy linked to ASF1B fits the four mechanisms above. Prostate, testicular, and ovarian cancers are treated separately here: their evidence is either too preliminary to assign to a core mechanism or derives from clinical contexts that those sections do not cover.

In prostate cancer (PCa), ASF1B has been identified as a key hub gene associated with disease progression through systems biology analysis.^90^ Its expression increases with disease progression and has been established as an independent prognostic marker for predicting postoperative biochemical recurrence (BCR) and cancer-specific survival.^91^ These clinical associations are consistent with ASF1B's core function in replication-coupled chromatin assembly and DNA repair, which sustains proliferative capacity and genomic integrity in rapidly dividing tumor cells. The mechanistic link between ASF1B and PCa progression (particularly its relationship with androgen receptor signaling) remains largely undefined. More recently, the gut-microbiota metabolite indole-3-lactic acid was shown to suppress ASF1B transcription via AHR-dependent recruitment of the HDAC1/2-NuRD complex; this suppresses the ASF1B/ENO1 axis, enhancing CD8^+^ T-cell infiltration and M1 macrophage polarization, and synergizing with anti-PD-1 therapy in prostate cancer models (in vivo),^92^ although ASF1B itself has not been directly targeted as a drug in this disease.

In testicular tumors, the tumor-associated histone variant H3.5 directly interacts with ASF1A/B.^93^ This interaction opens a new direction for exploring the non-canonical function of ASF1B in regulating chromatin states through partner-specific histone variants. The evidence is limited to in vitro protein-interaction assays, and the functional relevance of this interaction in testicular tumorigenesis remains to be determined.

In ovarian cancer, particularly high-grade serous ovarian cancer (HGSOC), more than 5% of patients exhibit amplification of the ASF1B gene copy number.^94^ Given that immunotherapy efficacy in HGSOC is constrained by a heterogeneous microenvironment, whether ASF1B expression tracks immune subtypes such as “immune-inflammatory” and “immune-desert,” and whether it offers a usable entry point for therapy in this setting, remains to be determined.

**Table 1.** In vivo studies of ASF1B in cancer models.

Cancer type	Model	Intervention	Key findings	Ref.
Cervical cancer	Subcutaneous xenograft (mouse)	ASF1B knockdown	Suppressed tumor growth and induced apoptosis (CDK9 stabilization)	[51]
Cervical cancer (ceRNA axis)	Xenograft (mouse)	Modulation of circCDK17/miR-122-5p axis	In vivo validation of the axis; suppressed progression	[83]
Gastric cancer	Subcutaneous xenograft (mouse)	ASF1B knockdown	Suppressed tumor growth; mechanism undefined	[63]
Gastric cancer	Xenograft and organoids (mouse)	ASF1B knockdown	Increased CD8^+^ T-cell infiltration	[69]
Gastric cancer (liver metastasis)	Spleen-injection liver metastasis model (mouse)	ASF1B knockdown	Inhibited the ZDHHC9/PCBP1/SLC7A11 axis and restored ferroptosis, reducing liver metastasis	[73]
LUAD	Xenograft (mouse)	ASF1B knockdown	Inhibited tumor growth in vivo; reduced Ki-67 expression	[58]
NSCLC	Syngeneic Lewis lung carcinoma (C57BL/6)	ASF1B ablation	Restricted tumor growth; enhanced anti-PD-L1 efficacy (p53-related ferroptosis)	[82]
HCC	Subcutaneous tumor formation (mouse)	Modulation of LINC02732/miR-1291/ASF1B axis	Axis promoted tumor growth; ASF1B recruits p300	[84]
Colorectal cancer	Xenograft (mouse)	ASF1B silencing (±PI3K/AKT activator)	Suppressed tumorigenesis; PI3K activation partially restored phenotypes	[64]
AML	Mouse leukemia models	ASF1B overexpression; Asf1b/Tlk2 deficiency	Overexpression accelerated progression; deficiency delayed it	[57]
Bladder cancer	Mouse xenograft model	ASF1B suppression	Reduced cisplatin resistance; inhibited tumor growth	[62]
ccRCC (SETD2-deficient)	Xenograft metastasis model	Targeting ASF1A/ASF1B complexes	Reduced distant metastasis	[72]
Melanoma	Xenograft (mouse)	Restoration of miR-767-3p (targets ASF1B)	Suppressed melanoma progression in vivo	[89]
Prostate cancer	Xenograft (mouse)	Indole-3-lactic acid (suppresses ASF1B transcription)	Attenuated ASF1B/ENO1 axis; enhanced anti-PD-1 efficacy	[92]

### 3.2. The role of ASF1B in non-cancerous diseases

Beyond cancer, the pathogenic relevance of ASF1B is increasingly recognized in a range of non-malignant diseases. This broadened landscape stems from the same core functions (chromatin assembly, cell cycle regulation, and epigenetic maintenance) that underpin its oncogenic roles. Depending on the cellular context and the nature of the dysregulation, these fundamental activities can drive tissue-specific pathologies: aberrant differentiation in skeletal disorders, immune imbalance in autoimmunity, or impaired stress responses in metabolic and infectious diseases. In line with this notion, abnormal expression of ASF1B has indeed been observed in skeletal, immune, metabolic, and even infection-related diseases,^95‐100^ suggesting its potential role as a cross-disease regulatory node (Figure 4). The following sections summarize the current evidence for ASF1B involvement in these non-malignant conditions.

**Figure 4.**
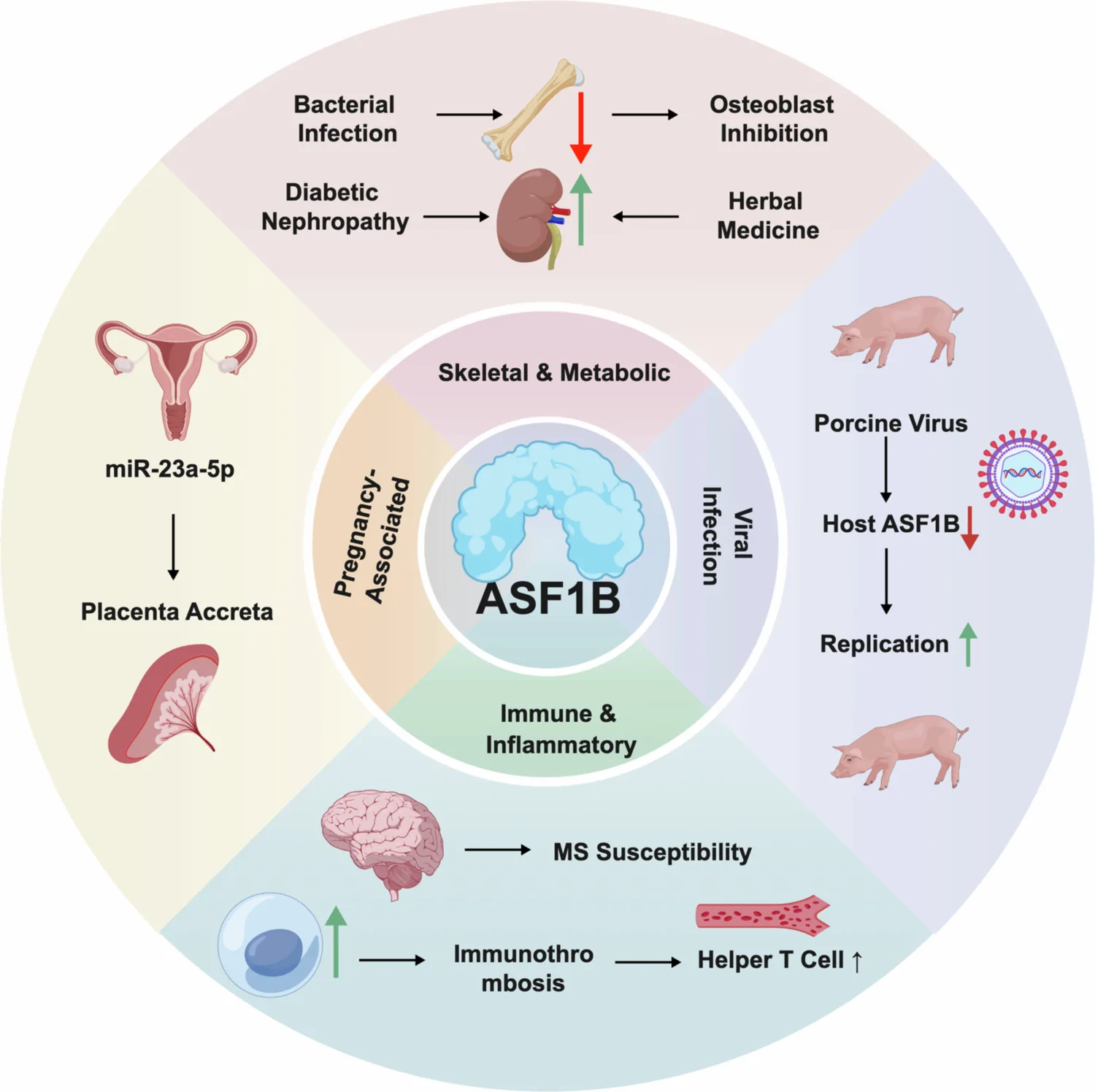
The hub network of ASF1B in bone metabolism, immunity, and infectious diseases. ASF1B participates in bone metabolism (osteogenesis inhibition associated with bacterial infection), metabolic disorders (upregulation in diabetic nephropathy, reversible by herbal intervention), and pregnancy-related conditions (peripheral-blood miR-23a-5p and its target genes, including ASF1B, as biomarkers for placental implantation disorders). In immune and inflammatory contexts, ASF1B is a susceptibility gene for multiple sclerosis and a hub gene correlating with helper T-cell infiltration in deep vein thrombosis. In host–pathogen interactions, porcine epidemic diarrhea virus (PEDV) suppresses host ASF1B expression to facilitate viral replication. Directionality of the depicted effects is schematic.

In skeletal and metabolic diseases, downregulation of ASF1B expression is directly associated with osteogenesis inhibition caused by bacterial infection.^95^ It is also abnormally upregulated in diabetic nephropathy models, and this abnormality can be reversed by traditional Chinese medicine intervention, suggesting its involvement in tissue damage under metabolic disorders.^96^ In immune and inflammation-related diseases, genome-wide analysis has identified it as a susceptibility candidate gene for multiple sclerosis.^97^ During the formation of deep vein thrombosis, it was identified as a hub gene in a transcriptomic analysis of deep vein thrombosis (DVT), with expression correlating positively with helper T-cell infiltration, suggesting its involvement in the mechanism of immune thrombosis.^98^ Among pregnancy-related diseases, miR-23a-5p and its target genes, including ASF1B, have been validated as peripheral-blood biomarkers for placental implantation disorders; the combination of miR-23a-5p with CHTF8 achieved an AUC of 0.869 for diagnosis and 0.856 for predicting adverse outcomes.^99^ In host-pathogen interactions, host ASF1B expression is suppressed during porcine epidemic diarrhea virus (PEDV) infection, suggesting a possible proviral role.^100^

Beyond cancer, then, ASF1B emerges as a multifunctional regulator of tissue homeostasis, immune response, and metabolic adaptation. Although each of these associations rests on one or two studies and the underlying pathways remain undefined, they converge on a plausible role for ASF1B as a regulatory node beyond oncology.

Table 2 summarizes the shared mechanisms and signaling pathways of ASF1B across diseases and indicates the strength of evidence for each entry.

**Table 2.** Mechanisms of ASF1B across diseases.

Mechanism	Key molecular events	Diseases	Evidence	Ref.
Cell cycle and proliferation	S-phase entry via CDK9/cyclin B1 axis; ASF1B interacts with CDK9; regulates PCNA and nucleosome assembly	HCC, Cervical, Gastric, AML, Breast, Bladder, LUAD, NSCLC	T+V; V+A; T+V; V+A; C+T; V+A; V+A; V+A	[51,52,57‐60,62,63,82]
Oncogenic signaling pathways	Activates PI3K/AKT/mTOR; induces c-Myc via CBP-mediated H3K56ac; transcriptionally activated by FOXM1; regulates oxidative stress via FOXM1-ASF1B-PRDX3 axis; activates PI3K/AKT and ERK1/2 by downregulating H2AC20	Gastric, Colorectal, Pancreatic, TNBC, ccRCC	T+V+A; T+V+A; V+A; V; C+V	[63‐65,67‐71]
Genomic maintenance, DDR and drug resistance	Participates in DSB repair (53BP1-RIF1); activates Chk1/Chk2; suppresses ferroptosis via ZDHHC9/PCBP1/SLC7A11 and p53-related pathways; SETD2 loss promotes metastasis via ASF1A/ASF1B-dependent chromatin remodeling	Pancreatic, Bladder, ccRCC, Rectal, Cervical, Gastric, NSCLC	C+V; V+A; V+A; C; V+A; T+V+A; V+A	[5,[51,[62,[70‐75,[82]
Tumor immune microenvironment	Correlates with infiltration of CD8^+^ T, CD4^+^ T, macrophages, Treg, B cells, neutrophils, and DCs; regulates PD-1/PD-L1; ASF1B knockdown increases CD8^+^ T-cell infiltration	HCC, Gastric, Thyroid, Glioma, Breast, NSCLC, Prostate	C+T+R; C+T+A; C+V; C; C+T; V+A; A	[60,73,76‐82,92]
Upstream regulatory network	ceRNA mechanisms (circCDK17/miR-122-5p, circUBAP2/miR-300, hsa_circ_0002024/miR-129-5p, SNHG3/miR-214-3p, LINC02732/miR-1291); H3K18-lactylation-p300 positive feedback; E2F1-driven transcription	Cervical, ccRCC, HCC, Pancreatic, Melanoma, TNBC, MM	V+A; C+V; V+A; T+V; V+A; V; V	[22,53,68,71,83‐89]
Non-oncological diseases	Regulates skeletal metabolism, immune response, metabolism, and viral infection	Skeletal diseases, MS, DKD, DVT, Viral infections	C+V; T; C+A; C+A; V	[95‐98,100]

Evidence levels are defined as follows: C, computational analysis only (public database or bioinformatic inference, without experimental validation); V, in vitro cell-based assays; A, in vivo animal models; T, clinical tissue validation (e.g., immunohistochemistry, qPCR); R, retrospective analysis of treated patient cohorts. Combinations indicate that multiple evidence types are available. Note on disease overlap: some diseases (e.g., gastric cancer, HCC, LUAD) appear in more than one mechanism category because ASF1B exerts pleiotropic effects through distinct molecular pathways in the same cancer type; this table is organized by mechanism rather than by cancer type. Limitations: the majority of the evidence summarized here is preclinical; prospective clinical validation of ASF1B as a diagnostic, prognostic, or therapeutic biomarker is largely lacking, and the immune microenvironment associations are predominantly computational inferences requiring experimental confirmation.

## 4. Clinical translation of ASF1B: diagnostic and therapeutic potential

ASF1B's clinical translation, in the strictest sense, remains minimal: to our knowledge, no ASF1B-targeted drug is approved, no ASF1B-directed agent has entered clinical trials, and no ASF1B assay is used in routine diagnosis. The nearest to clinical application is prognostic assessment from tumor tissue, supported by retrospective cohorts; everything therapeutic remains preclinical.

### 4.1. ASF1B as a diagnostic and prognostic biomarker

Computational analyzes of large public datasets have consistently identified ASF1B as a prognostic marker across multiple cancer types. A pan-cancer analysis of 33 human cancers found that ASF1B is highly expressed in 22 cancer types and is associated with poor prognosis in several major malignancies.^101^ However, most of these associations are derived from TCGA and GEO datasets without independent prospective validation.

Several cancer types have progressed beyond computational prediction to clinical tissue validation. In hepatocellular carcinoma (HCC), high ASF1B expression was associated with worse overall survival and progression-free survival in a training set (TCGA) and an independent validation set of 141 HCC patients.^76^ Multivariate analysis confirmed ASF1B as an independent prognostic factor; immunohistochemistry validated ASF1B protein expression in patient tissues. In breast cancer, qPCR and immunohistochemistry confirmed ASF1B overexpression in 67 tumor tissue samples compared with normal tissues, and high ASF1B was associated with reduced overall and recurrence-free survival and with HER2-positive status.^60^ In gastric cancer, a prognostic nomogram incorporating ASF1B expression, age, and clinical stage achieved an AUC of 0.925 for predicting survival, based on TCGA data and retrospective patient cohorts.^79^ By contrast, the evidence in glioma remains computational: analysis of TCGA, GTEx, and CGGA datasets identified ASF1B as an independent predictor of overall survival (HR = 1.573, 95% CI: 1.053–2.350, *p* = 0.027).^80^

A diagnostic AUC of 0.990 has been reported for ASF1B in pancreatic cancer.^102^ This value was derived from public GEO datasets, without an independent clinical validation cohort, and should be regarded as a database-level estimate rather than a clinically validated assay.

ASF1B has also been explored as a predictor of immunotherapy response. The relevant associations—positive in the Imvigor210 urothelial cancer cohort^76^ and in a retrospective gastric cancer series, negative in male thyroid cancer patients—are retrospective analyzes of treated cohorts, not prospective trials, and none has been validated prospectively with standardized immune monitoring (detailed in Section 3.1.4).

Across this literature, most studies are retrospective and database-derived, the evidence rests predominantly on mRNA expression, and standardized assays and cutoff values for clinical application are yet to be established.

### 4.2. ASF1B as a therapeutic target: current status and prospects

No ASF1B-specific small-molecule inhibitor currently exists. The reason is structural: the inhibitor-binding residues of ASF1B are highly conserved in ASF1A, and every compound reported so far (the pyrimidine-trione derivatives and the forthcoming family-level compounds) and the foldamer peptides—inhibits the ASF1 family rather than ASF1B alone.^34^ ^,^ ^103^ Both classes remain in vitro tools, with no isoform selectivity and no pharmacological data in animals. The target itself, however, is well validated: depleting ASF1B genetically suppresses tumor growth in cervical cancer xenografts^51^ and across the models reviewed in Section 3.1. The bottleneck in developing ASF1B therapy is therefore chemical rather than biological.

If a selective compound becomes available, the preclinical evidence points to two settings in which it could first be used. The first is biomarker-selected treatment. Tumors that have lost ASF1A may depend on ASF1B alone. ASF1A performs an NHEJ function that ASF1B cannot replace. Homozygous ASF1A deletion is reported in 13% of diffuse large B-cell lymphoma, 10% of prostate adenocarcinoma, 8% of pancreatic adenocarcinoma, and 3% of skin cutaneous melanoma,^104^ and the two paralogs are synthetic-lethal partners in cell-line screens.^105^ SETD2-deficient tumors likewise depend on ASF1-family complexes, and disrupting these complexes reduces metastasis in xenograft models.^72^ Tumors driven by TLK or FOXM1 could be targeted indirectly through these upstream nodes.^55^ ^,^ ^57^ ^,^ ^68^ ^,^ ^106^ Each of these settings offers a companion biomarker that could select patients for treatment. The second setting is combination therapy. ASF1B inhibition enhances cisplatin sensitivity, with in vivo proof-of-concept in bladder cancer.^62^ It also supports checkpoint blockade in preclinical models: ASF1B knockdown increased CD8^+^ T-cell infiltration in gastric cancer xenografts and organoids,^69^ and ASF1B ablation improved the efficacy of anti-PD-L1 therapy in a syngeneic NSCLC model^82^; the evidence for the immunotherapy setting rests on a small number of studies. Retrospective analyzes of treated cohorts link high ASF1B expression to better responses to immunotherapy,^76^ ^,^ ^79^ with one negative, sex-specific exception^81^; these associations could inform patient selection in trials but are not yet predictive on their own.

Outside oncology, functional studies show that ASF1B promotes human pancreatic *β*-cell proliferation, suggesting a potential route toward type 2 diabetes therapy^28^; antiviral applications of family-level inhibitors remain conceptual (Section 2.4). Reaching the clinic will require three steps: isoform-selective compounds, biomarker-guided trials in the tumor settings described above, and validation with pharmacological inhibitors rather than genetic tools (Figure 5).

**Figure 5.**
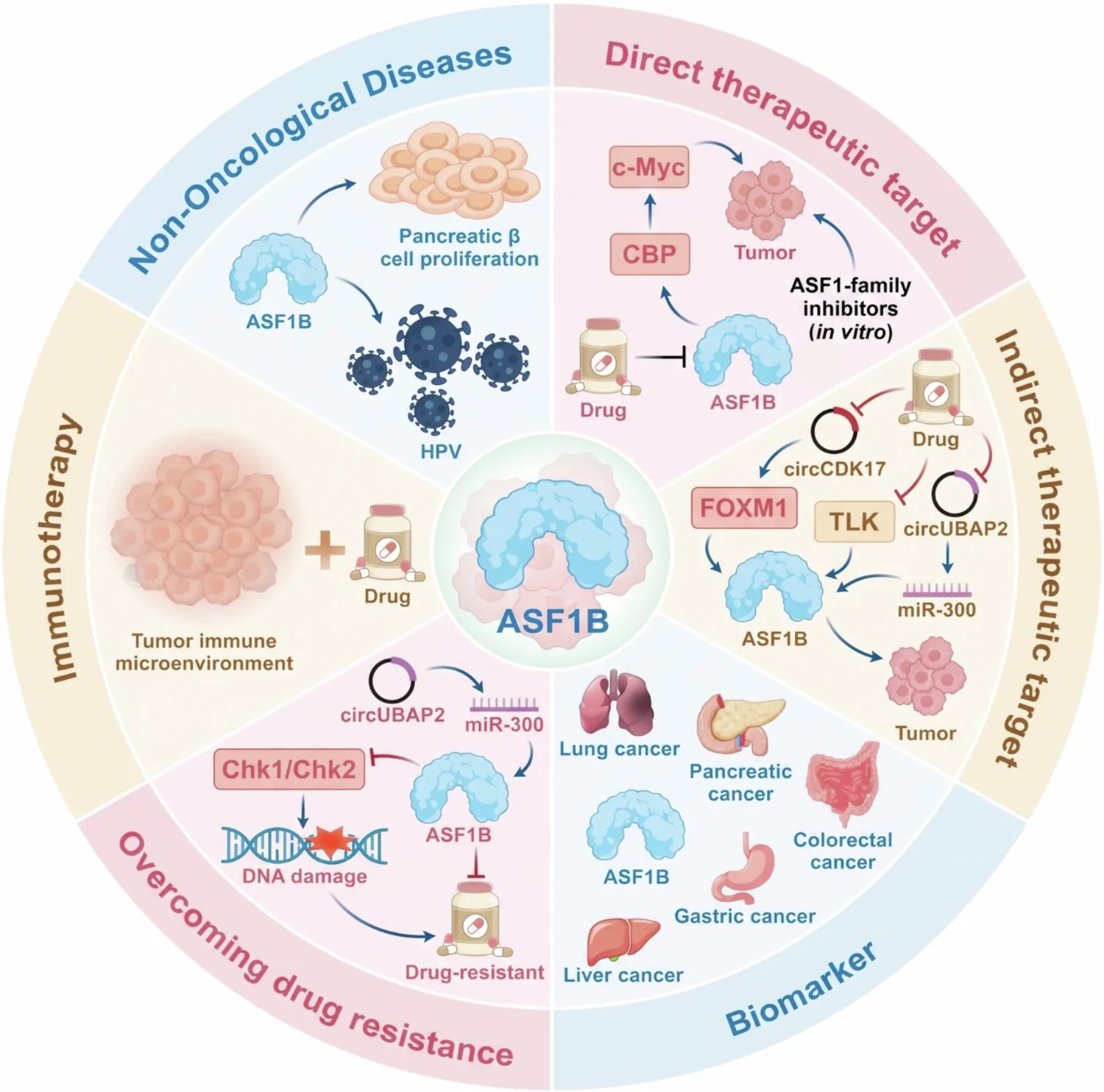
Translational landscape of ASF1B. This circular map summarizes the diagnostic and therapeutic applications of ASF1B reviewed in Section 4. The upper-right segment depicts direct targeting, which is currently achievable only through ASF1-family inhibitors active in vitro or genetic depletion, together with the CBP/c-Myc axis. The middle-right segment shows indirect strategies acting on upstream regulatory inputs: FOXM1 and TLK (transcriptional and kinase inputs, respectively) and drug-targetable ceRNA axes (circCDK17/miR-122-5p and circUBAP2/miR-300, with the drug icons shown). The lower segments summarize combination strategies to overcome drug resistance (Chk1/Chk2 checkpoint activation and DNA damage) and modulation of the tumor immune microenvironment for immunotherapy. The bottom-right segment lists cancers in which ASF1B serves as a diagnostic and prognostic biomarker (lung, pancreatic, colorectal, gastric, and liver cancers). The upper-left segment illustrates non-oncological applications, including the promotion of pancreatic *β*-cell proliferation and antiviral strategies targeting viral hijacking of ASF1 family proteins (e.g., HPV). Drug icons denote preclinical strategies only (genetic depletion or family-level ASF1 compounds), and none has entered clinical trials.

## 5. Critical view and future directions

First, ASF1B promotes malignancy through several distinct routes (cell cycle progression, oncogenic signaling, genome maintenance under treatment pressure, and immune microenvironment remodeling) rather than through a single mechanism, and these routes are controlled by an upstream network of ceRNA axes and transcription factors. Second, the evidence behind ASF1B varies greatly in strength and depends on context: ASF1B is consistently upregulated in most solid tumors but downregulated in multiple myeloma, and its value as an immunotherapy marker differs between cancers and between sexes. Third, ASF1B functions well beyond oncology, in erythropoiesis, early development, and host-pathogen interactions. These findings establish ASF1B as a genuine disease hub whose role is real but unevenly supported.

Two issues must be resolved before ASF1B can move toward clinical application. The first is evidentiary: most associations between ASF1B and prognosis, immune infiltration, or treatment response remain computational, and in vivo functional validation is concentrated in a small number of cancer types; extending functional studies to the remaining cancers is the clearest next step. The second is translational: no ASF1B-specific inhibitor exists, the therapeutic window of systemic inhibition is undefined, and all existing compounds are family-level and preclinical. Until then, ASF1B remains a strong biological target awaiting chemical validation.

## Data Availability

Data sharing is not applicable to this article as no new datasets were generated or analyzed during the current study.
